# Supplementary material for: Cholinergic network modulation in disinhibited eating behavior
Source: Commun Biol. 2025 Sep 17;8:1347. doi: 10.1038/s42003-025-08716-2 (PMC12443961; doi:10.1038/s42003-025-08716-2)

Supplementary material

Cholinergic network modulation in disinhibited eating behavior

Swen Hesse[1,2,*] , Michael Rullmann[1,2], Tilman Günnewig[1,2], Eva Schweickert de Palma[1,2], Lara Burmeister[1,3], Max van Grinsven[1,3]; Franziska Zientek[1,2], Julia Luthardt[1], Mohammed K. Hankir[4], Philipp M. Meyer[1], Georg-Alexander Becker[1], Marianne Patt[5], Peter Brust[6,7], Burkhard Pleger[8], Michael Stumvoll[9,], Anja Hilbert[10], Matthias Blüher[9,11], Osama Sabri[1]

[1]Department of Nuclear Medicine, University Medical Centre, University of Leipzig, Leipzig, Germany. [2]Integrated Research and Treatment Center Adiposity Diseases, University Medical Center Leipzig, Leipzig, Germany. [3]Department of Anaesthesiology Pain and Palliative Medicine, Radboud University Medical Center Nijmegen, Nijmegen, The Netherlands. [4]School of Biochemistry and Immunology, Trinity College Dublin, Ireland. [5]Section Radiopharmacy, Department of Nuclear Medicine, University Hospital Augsburg, Augsburg, Germany. [6]Department of Neuroradiopharmaceuticals, Institute of Radiopharmaceutical Cancer Research, Research Site Leipzig, Helmholtz-Zentrum Dresden-Rossendorf (HZDR), Leipzig, Germany. [7]The Lübeck Institute of Experimental Dermatology, University Medical Center Schleswig-Holstein, Lübeck, Germany. [8]Department of Neurology, BG University Hospital Bergmannsheil, Ruhr-University Bochum, Bochum, Germany; [9]Medical Department III, Endocrinology, Nephrology, Rheumatology, University Medical Center Leipzig, Leipzig, Germany; [10]Department of Psychosomatic Medicine and Psychotherapy, Integrated Research and Treatment Center Adiposity Diseases, Behavioral Medicine Research Unit, Leipzig, Germany. [11]Helmholtz Institute for Metabolic, Obesity and Vascular Research (HI-MAG) of the Helmholtz Zentrum München at the University of Leipzig and University Hospital Leipzig, University of Leipzig, Leipzig, Germany.

*e-mail: swen.hesse@medizin.uni-leipzig.de

	Page
Supplementary Fig. 1 Interregional connectivity of α4β2* nAChR availability	2
Supplementary Fig. 2 Individual V_T changes in different brain regions	4
Supplementary Fig. 3 Functional connectivity analyses	5
Supplementary Fig. 4 Correlation coefficients between V_T and beta estimates	7
Supplementary Fig. 5 Correlation coefficients between V_T and the Three-Factor Eating Questionnaire (TFEQ) sub-items	8
Supplementary Fig. 6 Visual food cues (examples)	9
Supplementary Fig. 7 Visual analog scale (VAS)	11
Supplementary Fig. 8 Group- and condition-specific correlation coefficients between changes of VAS assessments, V_T and fMRI beta estimates as well as TFEQ	12
Supplementary Fig. 9 Group- and condition-specific correlations of VAS assessment before and after each scan with V_T	13

Supplementary Fig. 1 Interregional connectivity of α4β2* nAChR availability under rest and stimulus conditions across the brain. Heatmaps of interregional V_T correlative analysis in normal-weight controls (**a**, under resting condition, **b** under stimulus condition) and in individuals with obesity (**c**, rest, **d**, stimulus) with low (**e**, rest, **f**, stimulus) and with high disinhibited eating behavior (**g**, rest, **h**, stimulus) (Pearson`s correlation coefficients and scaled FDR-adjusted *p*-value) suggesting altered brain state dynamics in obesity, i. e., with a loss of significant correlations in a network including the prefrontal cortex (PFC) and the nucleus basalis of Meynert (NBM) when comparing normal-weight controls and individuals with obesity, respectively. Of note, the small cohort size did not result in a significant *p*-value (>0.05) despite high correlation coefficients.

a

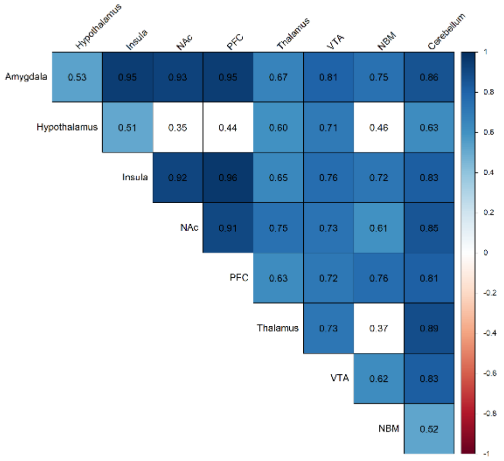

b

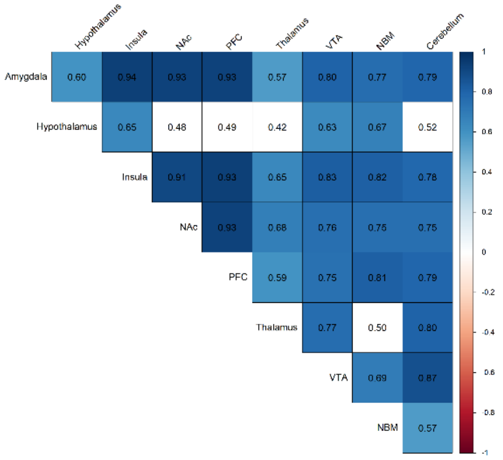

c

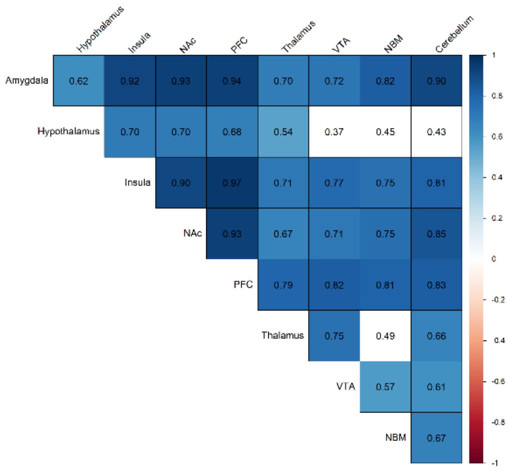

d

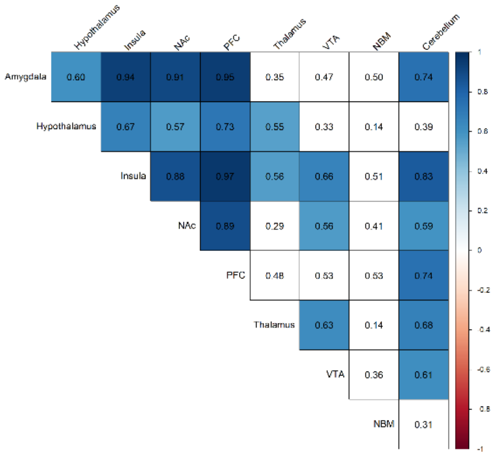

e

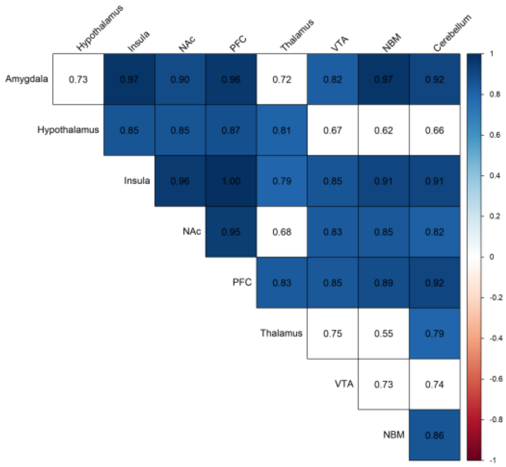
Hypothalamus Insula NAc PFC Thalamus VTA NBM Cerebellum
Amygdala 0.73 0.97 0.90 0.96 0.72 0.82 0.97 0.92
Hypothalamus 0.85 0.85 0.87 0.81 0.67 0.62 0.66
Insula 0.96 1.00 0.79 0.85 0.91 0.91
NAc 0.95 0.68 0.83 0.85 0.82
PFC 0.83 0.85 0.89 0.92
Thalamus 0.75 0.55 0.79
VTA 0.73 0.74
NBM 0.86
1 0.8 0.6 0.4 0.2 0 -0.2 -0.4 -0.6 -0.8 -1

f

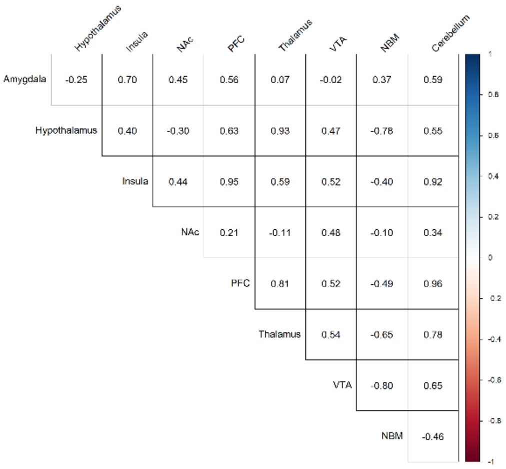
Hypothalamus Insula NAc PFC Thalamus VTA NBM Cerebellum
Amygdala -0.25 0.70 0.45 0.56 0.07 -0.02 0.37 0.59
Hypothalamus 0.40 -0.30 0.63 0.93 0.47 -0.78 0.55
Insula 0.44 0.95 0.59 0.52 -0.40 0.92
NAc 0.21 -0.11 0.48 -0.10 0.34
PFC 0.81 0.52 -0.49 0.96
Thalamus 0.54 -0.65 0.78
VTA -0.80 0.65
NBM -0.46

g

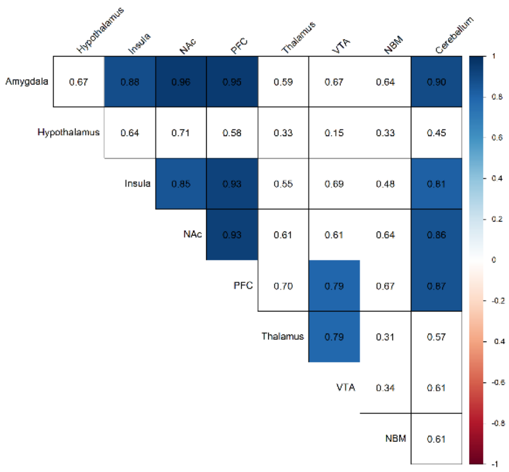
Hypothalamus Insula NAc PFC Thalamus VTA NBM Cerebellum
Amygdala 0.67 0.88 0.96 0.95 0.59 0.67 0.64 0.90
Hypothalamus 0.64 0.71 0.58 0.33 0.15 0.33 0.45
Insula 0.85 0.93 0.55 0.69 0.48 0.81
NAc 0.93 0.61 0.61 0.64 0.86
PFC 0.70 0.79 0.67 0.87
Thalamus 0.79 0.31 0.57
VTA 0.34 0.61
NBM 0.61

h

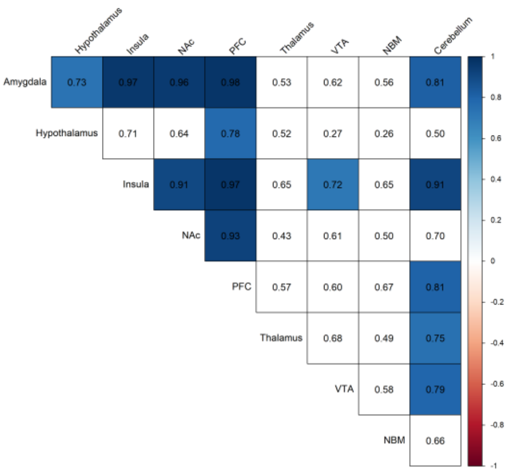
Hypothalamus Insula NAc PFC Thalamus VTA NBM Cerebellum
Amygdala 0.73 0.97 0.96 0.98 0.53 0.62 0.56 0.81
Hypothalamus 0.71 0.64 0.78 0.52 0.27 0.26 0.50
Insula 0.91 0.97 0.65 0.72 0.65 0.91
NAc 0.93 0.43 0.61 0.50 0.70
PFC 0.57 0.60 0.67 0.81
Thalamus 0.68 0.49 0.75
VTA 0.58 0.79
NBM 0.66
1 0.8 0.6 0.4 0.2 0 -0.2 -0.4 -0.6 -0.8 -1

Supplementary Fig. 2 Individual V_T changes in different brain regions in normal-weight individuals, individuals with obesity and high disinhibited eating behavior and individuals with low disinhibited eating behavior showing a high variability in the change of values among individuals with obesity when stimulus was added (NAc, nucleus accumbens; PFC, prefrontal cortex; VTA, ventral tegmental area; NBM, nucleus basalis of Meynert)

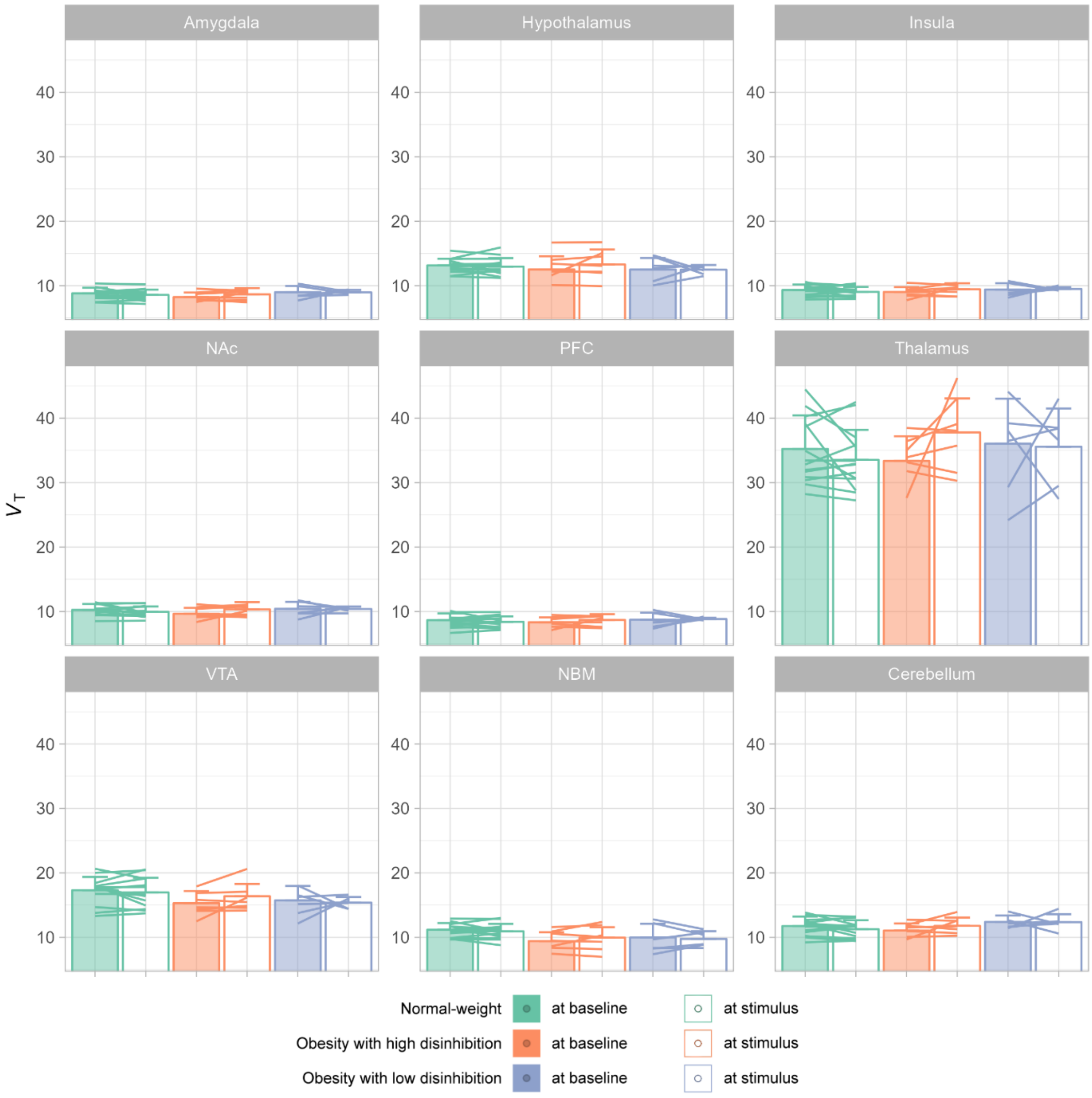

Supplementary Fig. 3 Functional connectivity analyses in normal-weight controls and in individuals with obesity. Statistical parametric mapping of resting state < food-cue stimulation data using **a,** the thalamus as the seed region and the Hillmer-corrected thalamic V_T as a covariate ($p_{uncorrected}$ < 0.001; paired t test; *n* = 14 normal-weight controls, *n* = 13 obesity excluding those without two scans, task-fMRI failures and lack of sufficient blood sampling, which avoids V_T calculation), as well as the NBM, nucleus basalis of Meynert, and the VTA, ventral tegmental area (covariate: V_T; $p_{uncorrected}$ < 0.001) in a **b,** two-sampled *t* test, and in a **c,** paired *t* test showing strengthened connectivity between the VTA and parts of the interior frontal cortex bilaterally in obesity and between the NBM and the VTA in normal-weight controls. **d,** anatomical delineation of this strengthened connectivity between the NBM and the VTA in a local acetylcholine (ACh) circuit.

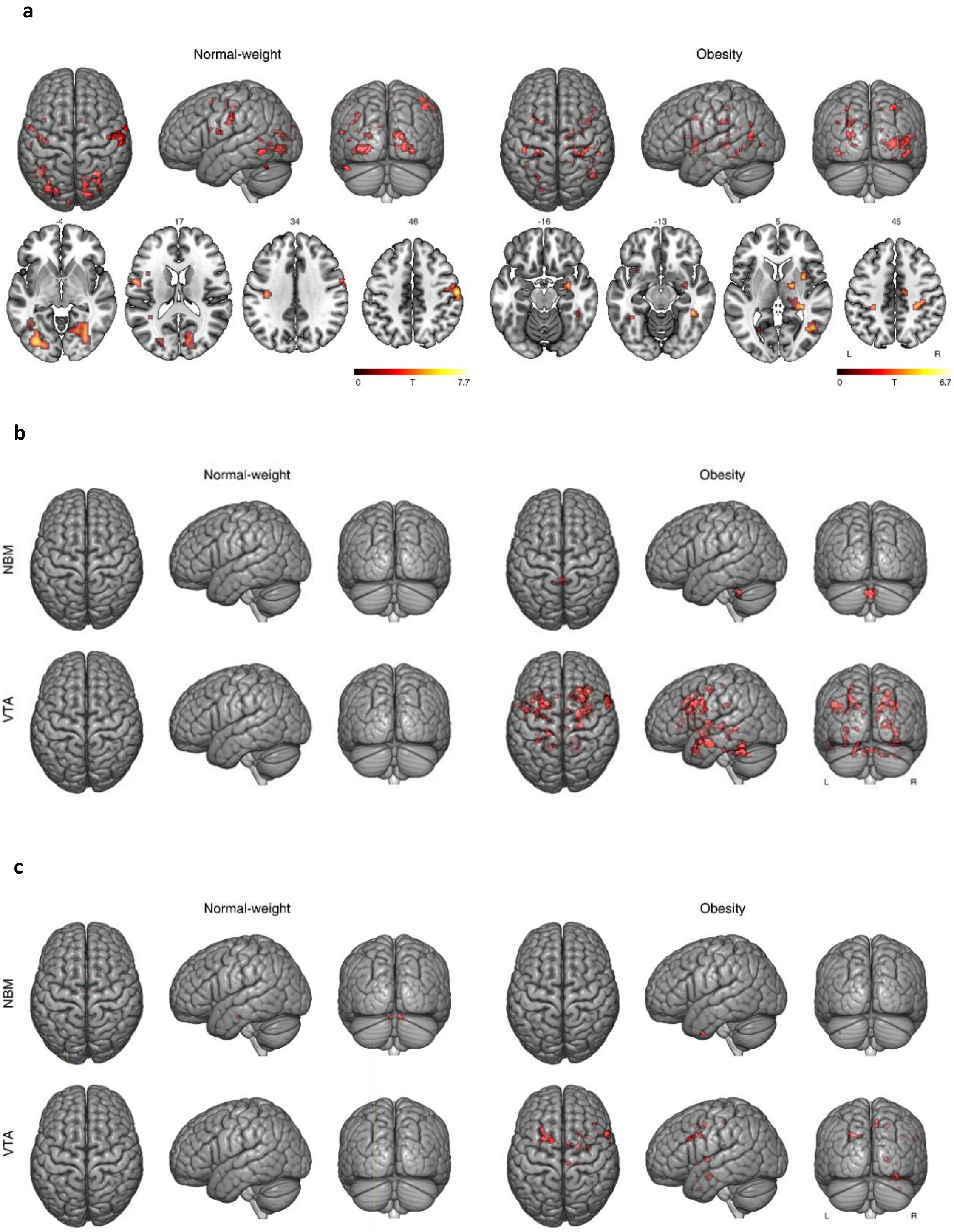

d

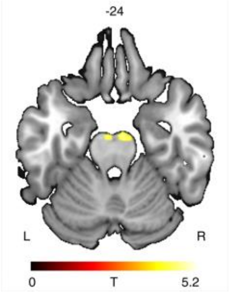

Supplementary Fig. 4 Correlation coefficients between V_T and beta estimates in clusters obtained from the network changes between resting state and visual food cue stimulation in individuals with obesity and normal-weight controls showing **a,** significant relationship between individual V_T and beta estimated in individuals with obesity but not in normal-weight controls applying the mask that is obtained from changes in functional connectivity strength in individuals with obesity. **b,** No significant interaction effect between both groups and their response to visual food cue stimulation (in comparison to resting state) in V_T ($F_{(1,58)}$ = 2.15, p = 0.152) can be found in those clusters even when solely including individuals with obesity and high disinhibited eating behavior ($F_{(1,45)}$ = 0.71, p = 0.4).

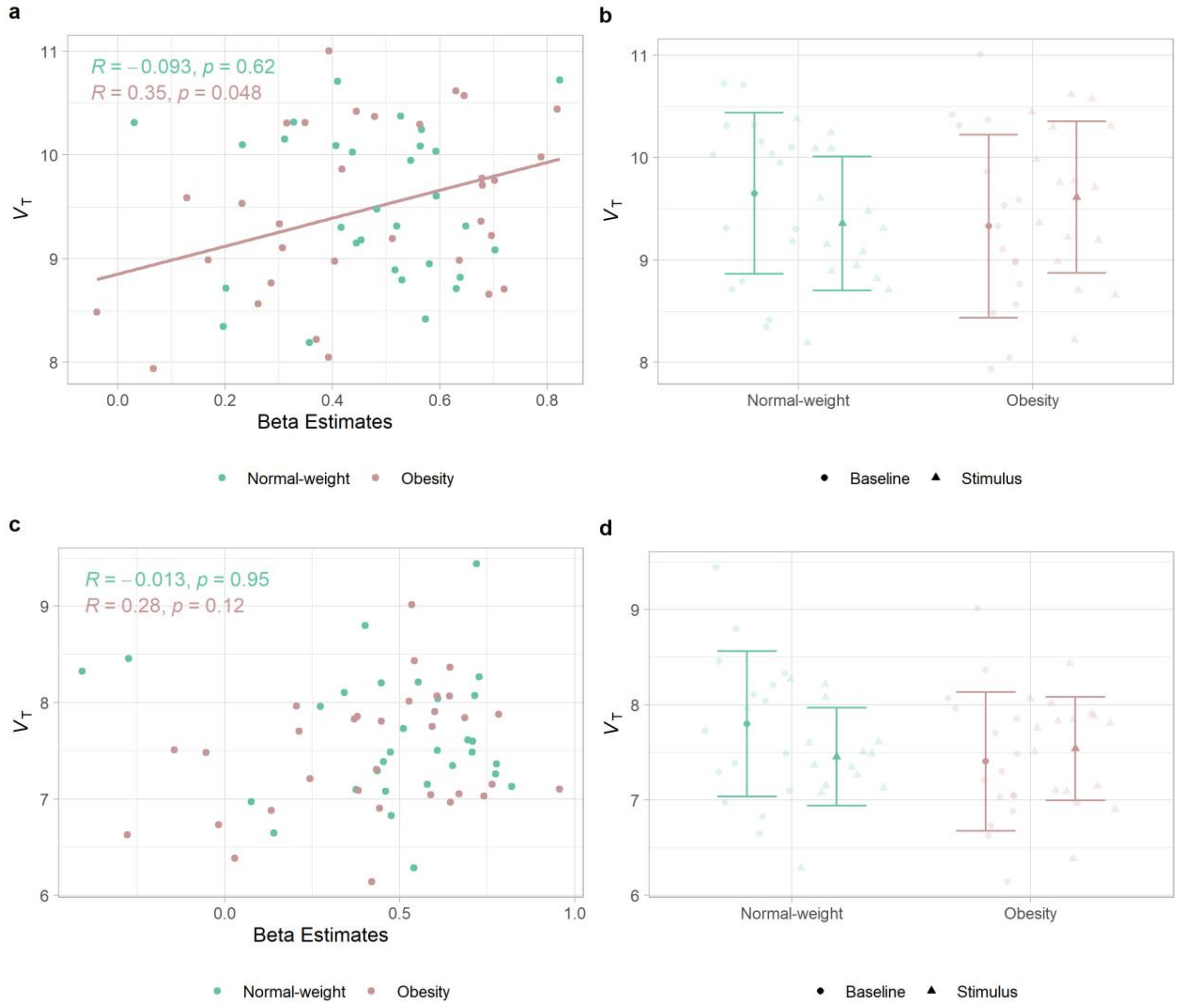

Supplementary Fig. 5 Correlation coefficients between V_T and the Three-Factor Eating Questionnaire (TFEQ) sub-items in **a**, normal-weight controls and in **b**, individuals with obesity. The different colors indicate the different directions of relationship (positive correlations in blue, negative correlations in red) with significance in the thalamus and in the hypothalamus vs. TFEQ 'cognitive restraint', p = 0.011 and p = 0.026, respectively, and with borderline significance in the cerebellum vs. TFEQ 'cognitive restraint' p = 0.0575. **c**, and **d**, Scatterplot showing the difference in the correlation of V_T versus TFEQ 'cognitive restraint' in the thalamus and the hypothalamus.

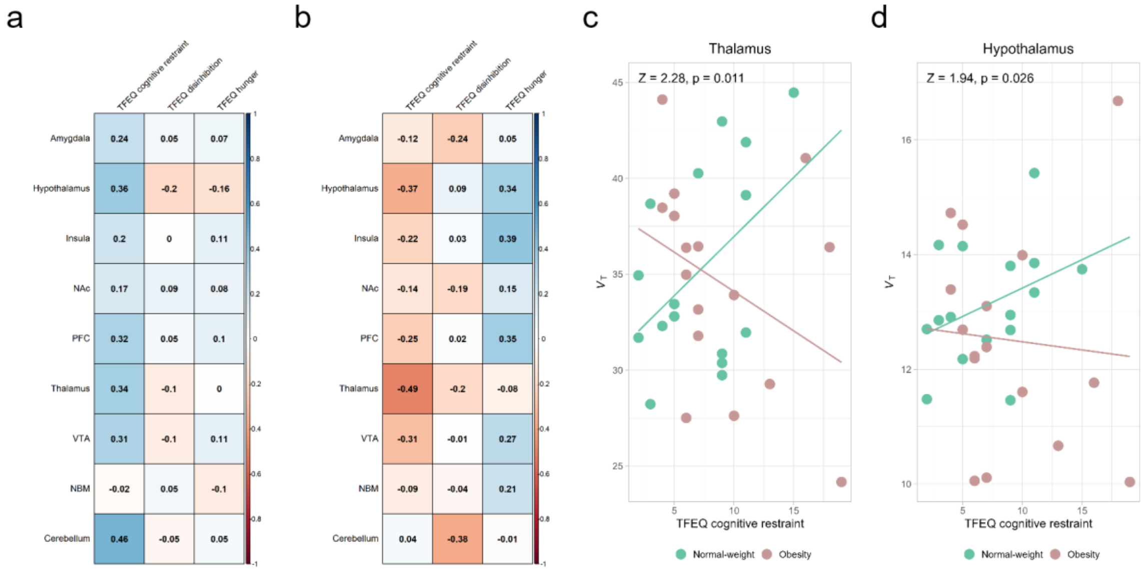

Supplementary Fig. 6 Visual food cues (examples) presented to the participants during the 2nd scan representing **a**, sweet (high-caloric) and **b**, savory (low-caloric) items

a

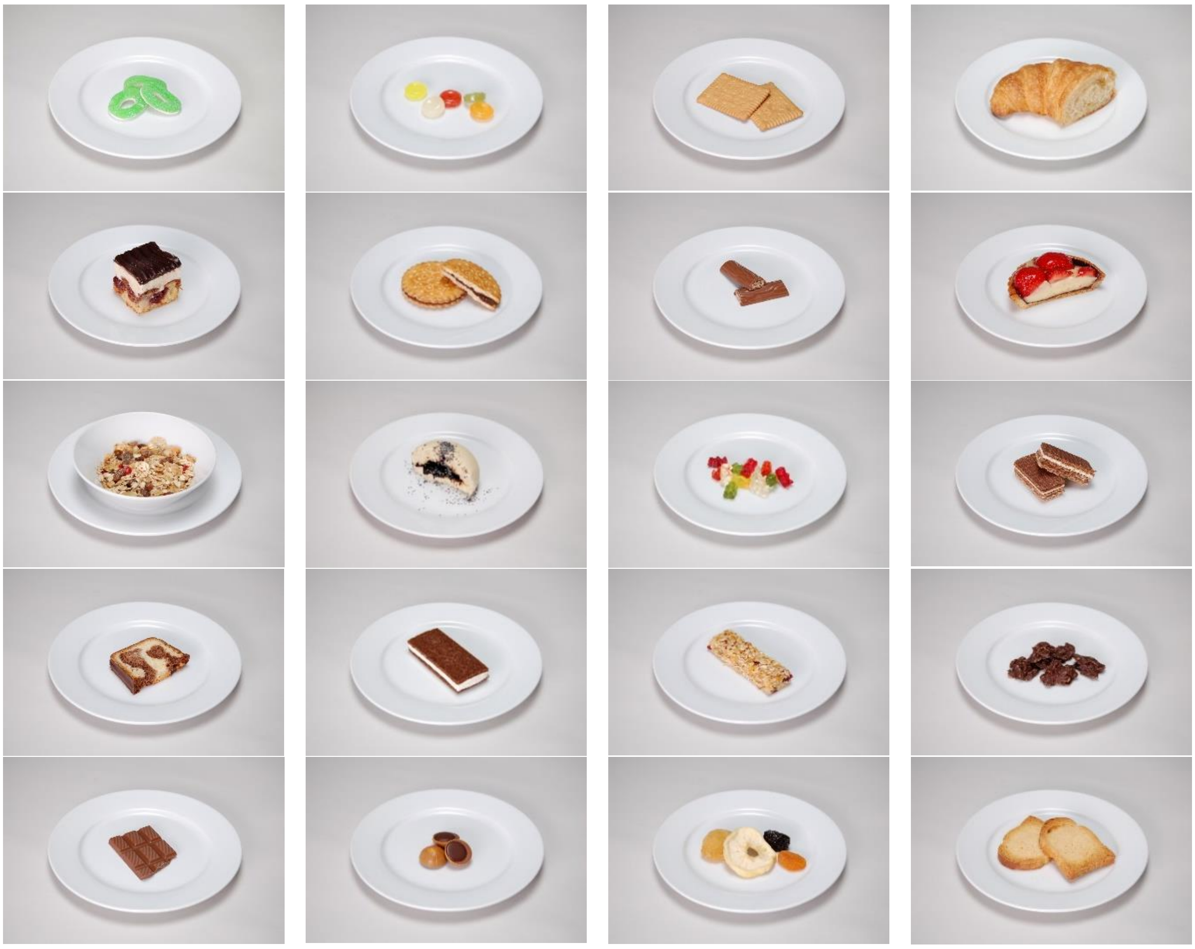

b

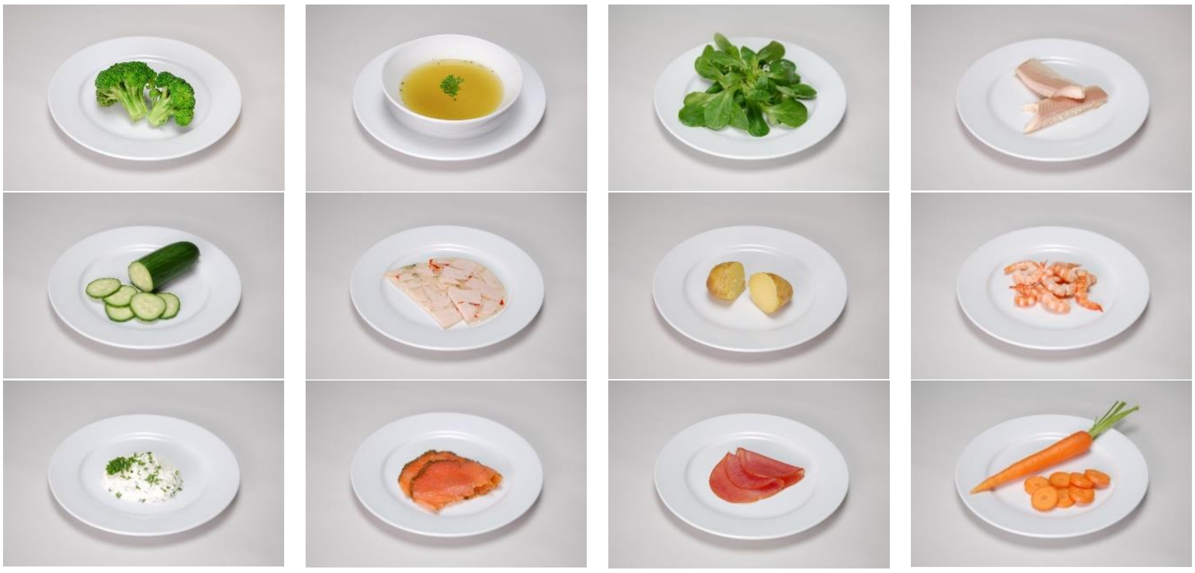

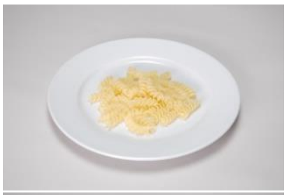
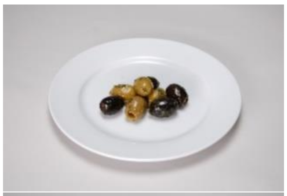
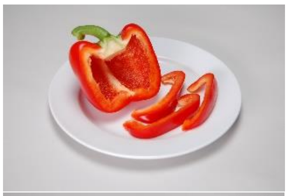
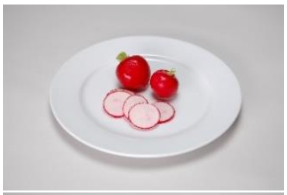
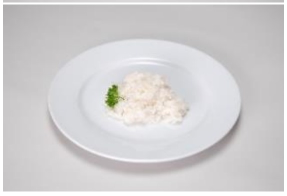
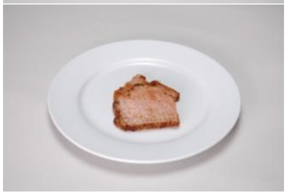
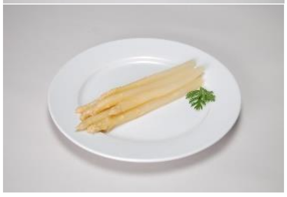
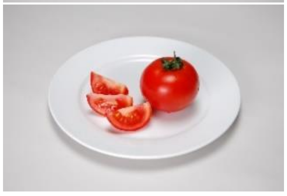

Supplementary Fig. 7 Visual analog scale (VAS) rating scale to assess the immediate feelings of hunger, satiety, wanting, disinhibition, and liking before and after each scan.

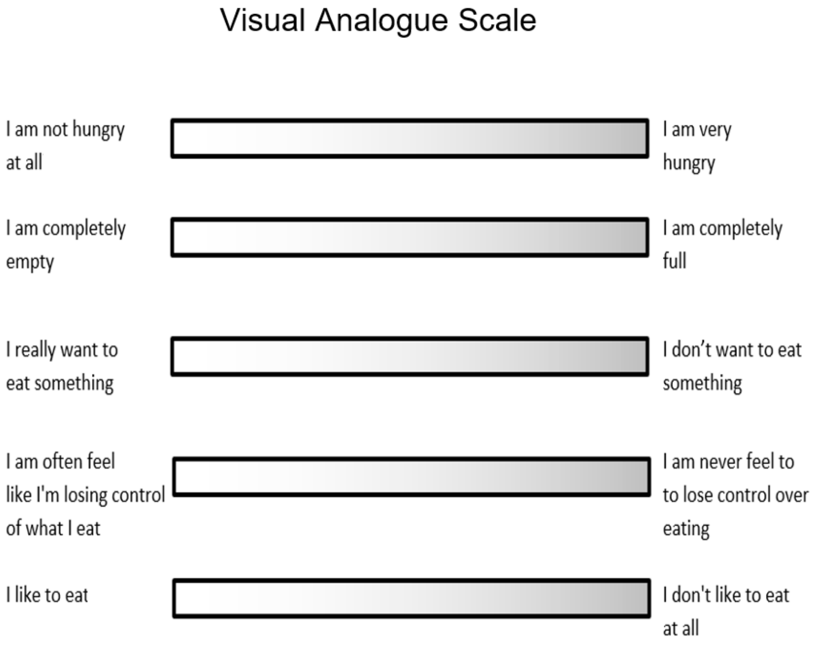

Supplementary Fig. 8 Group- and condition-specific correlation coefficients (Spearman rank correlation) between changes of visual analog scale (VAS) assessments, V_T and fMRI beta estimates as well as TFEQ in **a**, normal-weight controls and **b,** individuals with obesity ($\Delta VAS = VAS_{post\text{-}pre(stim)} - VAS_{post\text{-}pre(rest)}$). Changes in V_T was associated with changes in TFEQ 'cognitive restraint' (r = 0.021) while fMRI beta estimates showed correlation with changes in BMI in individuals with obesity (p = 0.03). V_T and fMRI estimates are extracted from the clusters when using the VTA as a seed (see Supplementary Fig. 3).

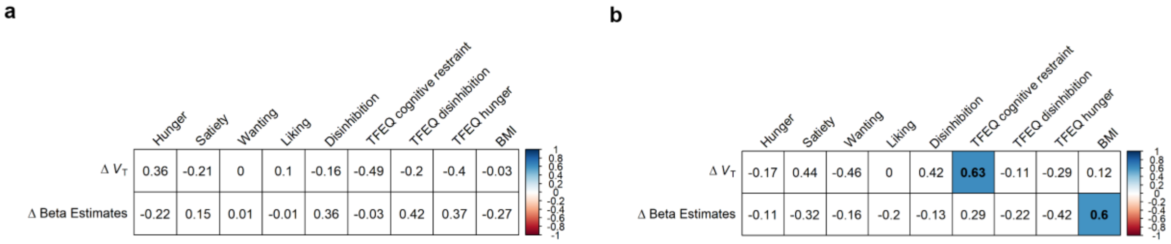

Supplementary Fig. 9 Group- and condition-specific correlations of visual analog scale (VAS) assessment before and after each scan with V_T under rest and under stimulus condition. a, In normal-weight controls, regional distribution volume V_T and VAS scores before resting state examination showed significant inverse correlation coefficients (Pearson) between VAS score for wanting and liking in the thalamus and the VTA before scanning under rest (p = 0.02, p = 0.04, and p = 0.011, respectively) and **b,** between V_T and VAS scores for liking in nearly all brain regions of interest before scanning with visual food cue stimulus (amygdala p = 0.005, insula p = 0.003, NAc p = 0.003, PFC p = 0.008, thalamus p = 0.041, VTA p = 0.001, cerebellum p = 0.011). **c,** In individuals with obesity, there was no association between V_T and VAS scores before scanning under rest while **d,** V_T values and VAS scores for liking did also correlate but inversely in the amygdala, the insula, the PFC, and the cerebellum (p = 0.014, p = 0.02, p = 0.049, and p = 0.019, respectively).

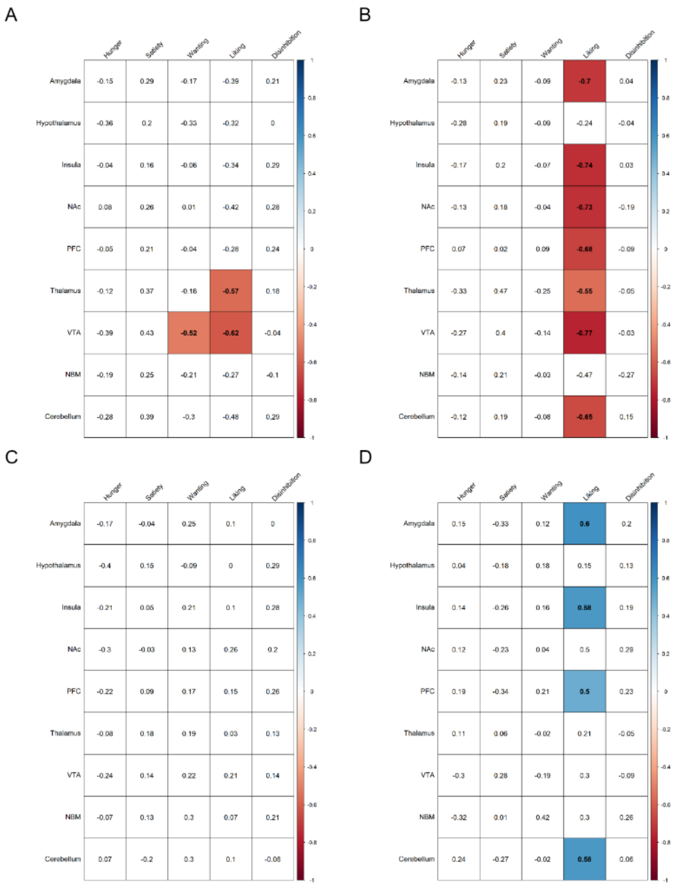

After scanning **e,** V_T and VAS scores for wanting showed significant inverse correlation coefficients (Pearson) in the amygdala r = -0.63, p = 0.009, the insula r = -0.59, p = 0.016; the NAc r = -0.66, p = 0.006), the thalamus r = -0.58, p = 0.018, the ventral tegmental area, VTA p = 0.001), and the cerebellum p = 0.01 but also between V_T in the VTA and VAS score for liking p = 0.03 in normal-weight controls. **f,** These correlations almost disappeared after stimulus condition in normal-weight controls (with only one significant correlation between V_T in the NAc and VAS score for liking (p = 0.046). **g,** In individuals with obesity, VAS score for disinhibition was associated with V_T in the hypothalamus p = 0.005 and in the thalamus with feelings of hunger p = 0.04 at rest **h,** which disappeared when visual food cues were presented while there was an association between cerebellar V_T and VAS score for liking during visual food cue stimulation p = 0.03. VAS score for liking after resting state examination showed significant higher values in normal-weight controls as compared with individuals with obesity and low disinhibited eating behavior and with individuals with high disinhibited eating behavior were registered (76.5 ± 13.2, 67.3 ±10.0, 65.9 ± 17.3, p = 0.024). No significant differences were found in the other VAS scores between the groups or before and after the scan.

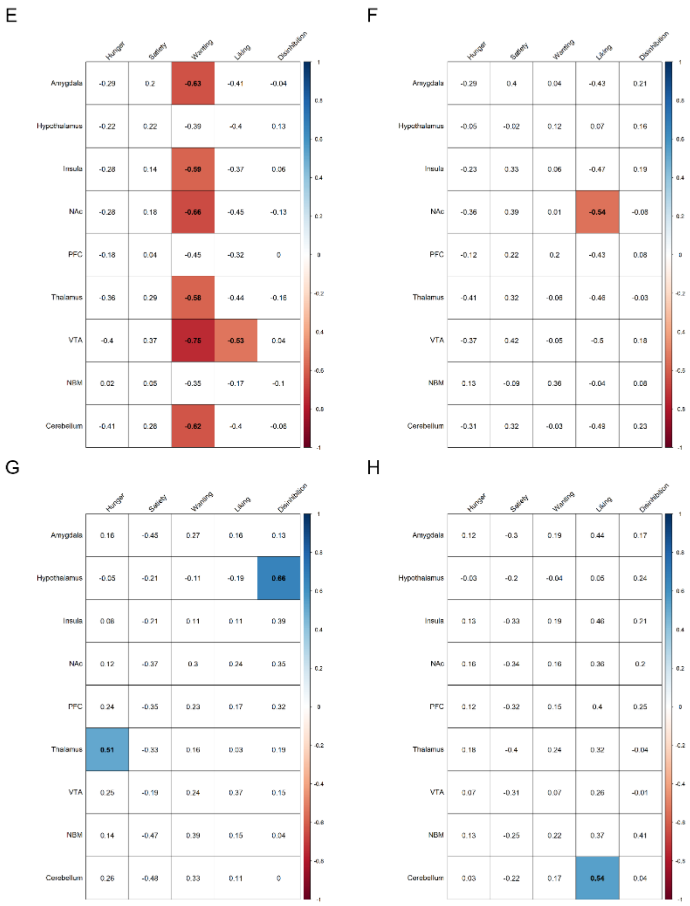

Supplement: Supplementary file 2 — Supplementary Information [file 42003_2025_8716_MOESM2_ESM.pdf]
