# Supplementary material for: Cholinergic network modulation in disinhibited eating behavior
Source: Commun Biol. 2025 Sep 17;8:1347. doi: 10.1038/s42003-025-08716-2 (PMC12443961; doi:10.1038/s42003-025-08716-2)
Supplement: Supplementary file 3 — Description of Additional Supplementary Files [file 42003_2025_8716_MOESM3_ESM.docx]

Description of Additional Supplementary Files

**File name:** Supplementary Data 1

**Description:** Source Data.
