# Supplementary material for: Cholinergic network modulation in disinhibited eating behavior
Source: Commun Biol. 2025 Sep 17;8:1347. doi: 10.1038/s42003-025-08716-2 (PMC12443961; doi:10.1038/s42003-025-08716-2)
Supplement: Supplementary file 5 — Reporting summary [file 42003_2025_8716_MOESM5_ESM.pdf]

nature portfolio

Corresponding author(s): COMMSBIO-25-3789-A

Last updated by author(s): August 8, 2025

Reporting Summary

Nature Portfolio wishes to improve the reproducibility of the work that we publish. This form provides structure for consistency and transparency in reporting. For further information on Nature Portfolio policies, see our Editorial Policies and the Editorial Policy Checklist.

Statistics

For all statistical analyses, confirm that the following items are present in the figure legend, table legend, main text, or Methods section.

n/a	Confirmed	
☐	☒	The exact sample size (*n*) for each experimental group/condition, given as a discrete number and unit of measurement
☐	☒	A statement on whether measurements were taken from distinct samples or whether the same sample was measured repeatedly
☒	☐	The statistical test(s) used AND whether they are one- or two-sided<br>*Only common tests should be described solely by name; describe more complex techniques in the Methods section.*
☐	☒	A description of all covariates tested
☒	☐	A description of any assumptions or corrections, such as tests of normality and adjustment for multiple comparisons
☐	☒	A full description of the statistical parameters including central tendency (e.g. means) or other basic estimates (e.g. regression coefficient) AND variation (e.g. standard deviation) or associated estimates of uncertainty (e.g. confidence intervals)
☐	☒	For null hypothesis testing, the test statistic (e.g. *F*, *t*, *r*) with confidence intervals, effect sizes, degrees of freedom and *P* value noted<br>*Give P values as exact values whenever suitable.*
☒	☐	For Bayesian analysis, information on the choice of priors and Markov chain Monte Carlo settings
☒	☐	For hierarchical and complex designs, identification of the appropriate level for tests and full reporting of outcomes
☐	☒	Estimates of effect sizes (e.g. Cohen's *d*, Pearson's *r*), indicating how they were calculated

Our web collection on statistics for biologists contains articles on many of the points above.

Software and code

Policy information about availability of computer code

Data collection	No software was used for data collection.
Data analysis	We used Matlab (The Mathworks Inc., Natick, USA) and R (https://www.R-project.org/).

For manuscripts utilizing custom algorithms or software that are central to the research but not yet described in published literature, software must be made available to editors and reviewers. We strongly encourage code deposition in a community repository (e.g. GitHub). See the Nature Portfolio guidelines for submitting code & software for further information.

Data

Policy information about availability of data

All manuscripts must include a data availability statement. This statement should provide the following information, where applicable:

- Accession codes, unique identifiers, or web links for publicly available datasets
- A description of any restrictions on data availability
- For clinical datasets or third party data, please ensure that the statement adheres to our policy

The datasets generated and analysed during the current study are available from the corresponding author on reasonable request. The data are not publicly available due to their containing information that could compromise the privacy of the participants.

Research involving human participants, their data, or biological material

Policy information about studies with human participants or human data. See also policy information about sex, gender (identity/presentation), and sexual orientation and race, ethnicity and racism.

Reporting on sex and gender	For the demographic descriptions, the proportion of each sex was given. No sex-specific analysis is reported in this study.
Reporting on race, ethnicity, or other socially relevant groupings	No data on race, ethnicity or other relevant social groupings are provided in this study
Population characteristics	The characteristics of the population are listed in the 'Results' section, including the relevant covariates (age, body mass index).
Recruitment	The study participants were recruited during ecotrophological consults at the University Hospital or by using public postings.
Ethics oversight	The study was performed in accordance with the Declaration of Helsinki with the guidelines for Good Clinical Practice (GCP), approved by the local ethics committee of the Medical Faculty of the University of Leipzig and the Bundesamt für Strahlenschutz (BfS; Federal Office for Radiation Protection), and registered under DRKS00010927 at the Deutsches Register für klinische Studien (DRKS; German Registry for Clinical Studies).

Note that full information on the approval of the study protocol must also be provided in the manuscript.

Field-specific reporting

Please select the one below that is the best fit for your research. If you are not sure, read the appropriate sections before making your selection.

☒ Life sciences ☐ Behavioural & social sciences ☐ Ecological, evolutionary & environmental sciences

For a reference copy of the document with all sections, see nature.com/documents/nr-reporting-summary-flat.pdf

Life sciences study design

All studies must disclose on these points even when the disclosure is negative.

Sample size	For this pilot study, the number of cases was estimated based on the results of an earlier receptor studies using the same radiotracer. Since in the planned study a PET/MR measurement of the distribution volume will be performed twice on one patient (without and after stimulus, matched-pair study), we expect the measurement difference to be normally distributed with a standard deviation of 0.15 (assuming a test-retest accuracy of the PET/MR scanner of 10%).Therefore, in a paired t-test with at least 10 participants, we can reject the null hypothesis of a response difference of 0 with a power of 0.9 if the actual response difference is 0.17 or more. The type 1 error probability associated with this test of the null hypothesis is alpha=0.05 (Dupont 1990).
Data exclusions	We exclude those without two scans, task-fMRI failures and lack of sufficient blood sampling, which avoids calculation of distribution volumes as it is reported in Ext. Data 3.
Replication	This is a pilot study design using fMRI and PET to assess functional connectivity and $\alpha 4\beta 2^*$ nACh receptor availability at baseline and in response to food cues in humans with obesity and high or low disinhibited eating behaviour and in non-obese controls that do not include additional test-retest experiments.
Randomization	Randomization was not performed since this is an observational study withour intervention. Therefore, subjects were not randomized into outcome groups.
Blinding	This was an observational study. Thus, the investigators were not blinded to group allocation.

Reporting for specific materials, systems and methods

We require information from authors about some types of materials, experimental systems and methods used in many studies. Here, indicate whether each material, system or method listed is relevant to your study. If you are not sure if a list item applies to your research, read the appropriate section before selecting a response.

Materials & experimental systems

n/a	Involved in the study
☒	☐ Antibodies
☒	☐ Eukaryotic cell lines
☒	☐ Palaeontology and archaeology
☒	☐ Animals and other organisms
☐	☒ Clinical data
☒	☐ Dual use research of concern
☒	☐ Plants

Methods

n/a	Involved in the study
☒	☐ ChIP-seq
☒	☐ Flow cytometry
☐	☒ MRI-based neuroimaging

Clinical data

Policy information about clinical studies

All manuscripts should comply with the ICMJE guidelines for publication of clinical research and a completed CONSORT checklist must be included with all submissions.

Clinical trial registration	The study was registered under DRKS00010927 at the Deutsches Register für klinische Studien (DRKS; German Registry for Clinical Studies).
Study protocol	The study protocol was approved by the local ethics committee of the Medical Faculty of the University of Leipzig but not available for publicity.
Data collection	Data were collected from 2015 to 2020 and stored according to the requirements of the local ethics committee.
Outcomes	The study outcomes were neuroimaging-based measures as described in the 'Methods' section. The protocol further includes sociodemo-graphic data, psychiatric and medical anamnesis, body morphometry, as well as neuropsychologcial questionnaires.

Plants

Seed stocks	N/A
Novel plant genotypes	N/A
Authentication	N/A

Magnetic resonance imaging

Experimental design

Design type	Both task and resting state was applied.
Design specifications	Food cues were presented during the 2nd scan once an equilibrium was reached after 120 minutes post injection. For the presentation of food cues, a video projection system presented food pictures (Presentation, NeuroBehavioral Systems, Inc., Berkeley, USA) via a projector using a screen viewed through a mirror in the head coil. During the task, a set of visual food cues (N = 80) was presented in a randomized block design (high- and low-caloric food) with each cue being presented for a period of 3 seconds. A randomized jitter (between 1.8 - 7.8 s) was applied between each cue.
Behavioral performance measures	Next to and after each scan, a visual analogue scale (VAS) was used to obtain data on feelings of hunger, wanting, disinhibition, satiety, and taste displayed in Ext. Fig. Data 6. Participants were asked to rate these factors on a continuous scale which was later translated into numbers ranging from 0 (= not at all) to 100 (= extremely) by measuring the distance in mm with a precision ruler as shown in Ext. Data Fig. 5.

Acquisition

Imaging type(s)	We applied functional and structural sequences.
Field strength	3T
Sequence & imaging parameters	fMRI data using echo-planar imaging and structural MR imaging data (using T1-weighted MP-RAGE) were acquired alongside three-dimensional PET data acquisition with the following parameters: MP-RAGE (176 contiguous sagittal

slices with 1 mm thickness and no gap; repetition time (TR)/echo time (TE) = 1900/2.53 ms; inversion time (TI) = 900 ms; flip angle = 9°; field of view = 250 × 250 mm; matrix = 512 × 512; voxel size = 1.0 × 0.48 × 0.48 mm) and BOLD fMRI (600 echo planar imaging (EPI) volumes with a voxel size of 3 × 3 × 4.2 mm, TR = 2000 ms, TE = 30 ms, flip angle = 90°, and slice thickness of 3.5 mm).

Area of acquisition: Whole brain.

Diffusion MRI: ☐ Used ☒ Not used

Preprocessing

Preprocessing software: Co-registration and motion-correction procedures of PET and MR imaging data were performed using SPM12-software (Statistical Parametrical Mapping, Wellcome Trust Centre for Neuroimaging, University College London, UK).

Normalization: fMRI were spatially normalized to MNI space.

Normalization template: fMRI were spatially normalized to MNI space.

Noise and artifact removal: Motion correction was performed within SPM.

Volume censoring: Basic preprocessing steps included the removal of the first 10 time points.

Statistical modeling & inference

Model type and settings: For the seed-based fMRI analysis we build a general linear model on single-subject level. After applying the seed to extract the first Eigenvariate of the beta values across all voxels within the seed mask, the resulting individual time series was implemented within the same single-subject model as an additional non-interacting regressor to test for a positive correlation (i.e. strengthened connectivity) of the seed region throughout the entire brain.

Effect(s) tested: The individual statistical maps were entered into a group-level two-sampled t-test with the corresponding individual distribution volume of the seed region to assess differences in functional connectivity of the seed in relation to the individual distribution volume (i.e. interaction between fMRI and PET) between both scanning conditions, at rest and with visual stimulus.

Specify type of analysis: ☒ Whole brain ☐ ROI-based ☐ Both

Statistic type for inference (See Eklund et al. 2016): P < 0.001; two sampled t test

Correction: uncorrected

Models & analysis

n/a	Involved in the study
☐	☒ Functional and/or effective connectivity
☒	☐ Graph analysis
☒	☐ Multivariate modeling or predictive analysis

Functional and/or effective connectivity: Individual time series was implemented within the same single-subject model as an additional non-interacting regressor to test for a positive correlation (i.e. strengthened connectivity) of the seed region throughout the entire brain
